# Integration of a Mobile Node into a Hybrid Wireless Sensor Network for Urban Environments †

**DOI:** 10.3390/s19010215

**Published:** 2019-01-08

**Authors:** Carlos Alberto Socarrás Bertiz, Juan Jesús Fernández Lozano, Jose Antonio Gomez-Ruiz, Alfonso García-Cerezo

**Affiliations:** 1 Faculty of Engineering, Universidad de la Guajira, Riohacha 440001, Colombia; csocarras@uniguajira.edu.co; 2 Robotics and Mechatronic Lab, Andalucía Tech, Universidad de Málaga, 29071 Málaga, Spain; janto@uma.es (J.A.G.-R.); ajgarcia@uma.es (A.G.-C.)

**Keywords:** wireless sensor network, mobile sensors, hybrid wireless sensor networks, Cyber-Physical Systems, urban environment monitoring

## Abstract

Robots, or in general, intelligent vehicles, require large amounts of data to adapt their behavior to the environment and achieve their goals. When their missions take place in large areas, using additional information to that gathered by the onboard sensors frequently offers a more efficient solution of the problem. The emergence of Cyber-Physical Systems and Cloud computing allows this approach, but integration of sensory information, and its effective availability for the robots or vehicles is challenging. This paper addresses the development and implementation of a modular mobile node of a Wireless Sensor Network (WSN), designed to be mounted onboard vehicles, and capable of using different sensors according to mission needs. The mobile node is integrated with an existing static network, transforming it into a Hybrid Wireless Sensor Network (H-WSN), and adding flexibility and range to it. The integration is achieved without the need for multi-hop routing. A database holds the data acquired by both mobile and static nodes, allowing access in real-time to the gathered information. A Human–Machine Interface (HMI) presents this information to users. Finally, the system is tested in real urban scenarios in a use-case of measurement of gas levels.

## 1. Introduction

Wireless sensor networks (WSNs) allow for the acquisition of information when proximity from the sensors to the phenomenon and persistence over time is required [1,2]. WSNs have been applied to problems like logistics, traceability, emergency operations or urban environments [3,4,5,6,7]. In emergency response, WSNs can provide close monitoring with low cost, flexibility and scalability [8,9], as well as way of integrating robot into rescue teams [10], as a step towards Cyber-Physical Systems (CPS) for first response [11,12,13]. In urban environments, WSNs can help monitoring power systems, solid waste or water facilities [14]. But traffic has attracted most of attention, since it is generally considered as one of the main problems affecting the health of city population [15]. The number of vehicles has grown notably in recent decades; as has pollution [16]. Pollution data are usually measured using an infrastructure of fixed sites. Such an infrastructure is limited, expensive and static. As a result, pollution and air quality data are limited [17,18]. WSNs, on the contrary, are flexible and dynamic, and can be easily adapted to rural or urban environments. These features have made WSNs a valuable alternative for the way Intelligent Transportation Systems (ITSs) are implemented nowadays. ITSs are a way of improving transportation efficiency and safety [19], but also to save energy and to reduce emissions of vehicles [20] through a better management of available resources. But one of the obstacles to the implementation of new transport strategies is the limited amount of information on urban traffic. WSNs provide a mean to improve the amount and quality of the data available for planning and management in ITSs [21,22]. 

The effectiveness of WSNs depends strongly on the network coverage and connectivity provided by the sensor deployment [23,24]. Even if sensor nodes are usually easy to install, once they have been placed they only way to improve the information they acquire is to add new nodes, or to re-deploy the existing ones. Thus, the strategy to select the locations of the nodes becomes a key feature for the performance of WSNs [25,26,27]. 

A complementary strategy is to provide mobility to the sensor nodes, for example by including a method for replacing the sensor nodes if they present a malfunction [28]. Another option is to mount the sensor nodes onboard vehicles or mobile robots [29,30]. This approach is valid not only for urban applications, but also for other use-cases, like disaster robotics [6]. Mobility of the sensor nodes can be found in mobile ad hoc networks (MANETs), defined as a set of wireless mobile nodes characterized as self-adaptive and infrastructure-less [9]. In vehicular ad hoc networks (VANETs), or in general, in vehicular sensor networks (VSNs), moving vehicles and infrastructure become nodes of a dynamic network [20]. In general, a WSN including both static and mobile sensor nodes can be considered a Hybrid Wireless Sensor Networks (H-WSNs). Several authors have proposed VSNs for real-time data acquisition and monitoring [8,31,32,33,34,35], including solutions like cloud computing [36], fog-based event monitoring [37] or high resolution maps in urban environments [38].

The existence of mobile nodes in VSNs (or, in general, in H-WSNs) can make difficult to ensure coverage. The development of a H-WSN must pay special attention to this feature [39]. 

A classical approach to design a WSN considers a flat architecture. However, this kind of network does not allow for applications with mobile nodes [40], since a constant reconfiguration of the network is then required [29]. A hierarchical architecture can improve performance in these cases [41,42,43], decreasing delays, while improving the reliability of data transmission and connectivity of the network [40]. However, special attention must be paid to the organization of the network [44,45]. For instance, the use of a mobile sink can allow for a longer lifetime of sensor nodes avoiding multi-hop data collection [46].

Another important issue in H-WSNs is network overhead [47]. Routing protocols have to be adapted to limited time communication links [32,48,49]. A proposed solution is using position aware routing protocols [47,50]. Another proposal is dynamic routing [8]. While using MANETs can be a comparatively simple solution [32,50], the need for frequent topological updated undermines energy consumption [51]. Security and privacy are also concerns when using MANETs [52], as well as adaptability and scalability [53]. A more robust option is using H-WSNs, although the location of mobile and static nodes has to be carefully designed, particularly for the base stations. An alternative is employing mobile base stations or mobile sinks, and dynamic relocation [42,54]. Optimization based clustering has been also proposed by several authors [46,55,56]. As for security and reliability, H-WSNs allow for reliable and timely data communication, for instance using dynamic congestion control schemes [57].

This paper presents the integration of a mobile node into a wireless sensor network for urban environments, constituting a hybrid wireless sensor network. The mobile node has a modular architecture, allowing different sensors to be included for every deployment, like gas, temperature, humidity or GPS location. The information provided by the sensors is stored in a local database, and synchronized with an external database located in a server. The synchronization reduces the need for multi-hop routing, thus ensuring reliable and timely data delivery. A Human–Machine Interface (HMI) allows for real-time monitoring of the information from the sensors, including location of the measurements. 

The main contributions of this paper are: a) the implementation of a mobile sensor node with a modular architecture which enables the installation of different sensors depending on mission needs; b) the integration of the mobile node into a static wireless sensor network, without the need for multi-hop routing or dynamic relocation methods, resulting in a hybrid wireless sensor network of improved range and flexibility respect to the original; c) experiments validating the mobile node and its integration, including real-time availability and presentation of the gathered information. 

This paper is structured as follows: after this introduction, Section 2 describes the original wireless sensor network serving as the basis for the hybrid wireless sensor network. Section 3 presents the mobile sensor nodes and its integration into the hybrid wireless sensor network. Section 4 is devoted to the experiments performed to validate the system. Finally, Section 5 contains the conclusions. 

## 2. Description of the Static Wireless Sensor Network

### 2.1. System Architecture

**Figure 1:**
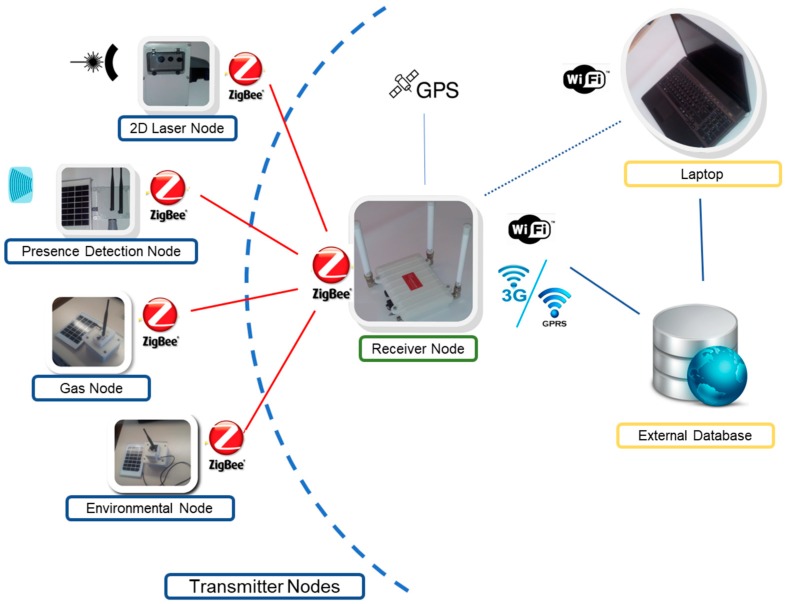
System architecture of the UIS static Wireless Sensor Network.

The system is based on a static wireless sensor network developed for urban applications, called Urban Information System (UIS) [7]. The UIS has a modular architecture to adapt the system configuration to the needs of the area of interest. Two different types of nodes were originally developed: Transmitter nodes and Receiver nodes. The basic configuration of the UIS platform comprises several Transmitter nodes and at least one Receiver node. The Transmitter nodes collect urban environment information such as Bluetooth MACs, number of vehicles crossing the ultrasound beam, gases concentration like NO_x_, CO, CO_2_, O_2_, NH_3_, VOC, light intensity, noise or dust. The UIS can be deployed with a different number of Transmitter nodes [58]. The Receiver node configures and manages the UIS network. Once the network has been set up, the Transmitter nodes carry out the data acquisition and processing. Then the data are sent to the Receiver node, where an internal database is updated and synchronized with the database in an external server (see Figure 1). A graphical Human–Machine Interface (HMI) presents data from the database related to their geographic position. A solar kit can be installed in all the nodes so that they can be independent from the electric power grid, and autonomous from an energy point of view. This way, deployment of the nodes gain flexibility.

The flexibility in the deployment is one of the advantages of WSNs compared with fixed infrastructure. Sensors can be positioned easily and in short time, but they can also be distributed to study a particular phenomenon. For instance, in the case of UIS, nodes can be deployed to estimate the origin-destination matrix (O-D matrix) for a particular set of points [17]. This matrix represents the number of vehicles going from origin i to destination j. To estimate this number, a relevant sample of the vehicles traveling from i to j has to be identified. The proposed UIS does so by positioning Bluetooth nodes nearby the points of interest [7]. These nodes collect the MAC address of Bluetooth devices within their range, and transmit them to the Receiver node. All this information is synchronized with the database in the server, and then the O-D matrix is estimated, given that the location of the Bluetooth nodes is known [23]. Thus, freedom to select the points where the transmitter nodes are deployed is key for this feature to be useful for traffic managers.

### 2.2. Implementation

The proposed system has been developed on the basis of hardware components provided by Libelium (Zaragona, Spain) [59]. The transmitter nodes share a basic module, called Waspmote V. 1.2 (Libelium, Zaragoza, Spain) The addition of an XBee Pro S2 communications module (Digi, Minnetonka, MN, U.S.A.) provides the capability of using protocols like ZigBee or DigiMesh. The Receiver node is based on a multiprotocol router named Meshlium, also from Libelium, configured to work with ZigBee, WiFi, Bluetooth, and 3G/GPRS protocols, and including a GPS. The link between the Transmitter nodes and the Receiver node has been implemented using the ZigBee protocol (2.4 GHz). It can transmit small information packages with a range of over 700 meters (depending on visibility conditions). Network setup is fast and simple, and so is adding new nodes.

As mentioned above, several Transmitter nodes have been developed, including different sensors. This way, a UIS deployment can include the following types of nodes:

- UIS Bluetooth node. It includes a BLUEGIGA WT12 Bluetooth module to the Waspmote V1.2 platform, along with the communications module XBee Pro S2, programmed to work with ZigBee wireless and Bluetooth 2.1 + EDR protocols simultaneously.
- UIS Ultrasound node. It adds an XL-MaxSonar-WR1 ultrasonic sensor (Maxbotix, Brainerd, MN, U.S.A.) to the initial configuration. These sensors operate at a frequency of 42 KHz, and reach the maximum range of 6 m with a sensitivity of 3.2 mV/cm to 3.3 V, or 7 m and a sensitivity of 4.9 mV/cm to 5.5 V.
- UIS Laser node. It is based on a Nano Pico ITX 1.2 GHz processor board (Via Technologies Inc., Taiwan) including 4 GB RAM DDR3 memory and a solid-state hard disk with a capacity of 60 GB. The laser sensor is a Hokuyo model UTM-30LX-EW (Osaka, Japan). It is intended to classify the types of vehicles crossing a given section.
- UIS Environmental Pollution node. It includes a dust sensor (GP2Y1010AU0F, Sharp, Osaka, Japan) a light intensity sensor (GL5528 photoresistor, Lida Optical&Electronic Co. Ltd., Henan, China) and a noise sensor (WM-61a, Panasonic, Osaka, Japan).
- UIS Gas node. It is composed of several gas sensors: O2 (SK-25, from Figaro, Osaka, Japan), O3 (MICS-2610, from E2V, Essex, U.K.), CO2 (TGS 4161, from Figaro), CO (TGS 2442, from Figaro), NH3 (TGS 2444, from Figaro), VOC (TGS 2600, from Figaro). Additional sensors include humidity (J808H5V5, from Jin Zon Enterprise Co. Ltd., Taiwan), atmospheric pressure (MPX4115A, from Motorola, Tokyo, Japan) and temperature (MCP9700/9701, from Microchip, Arizona, U.S.A.).
- GPS node. It includes a Jupiter N3 GPS module from Telit (London, U.K.).

**Figure 2:**
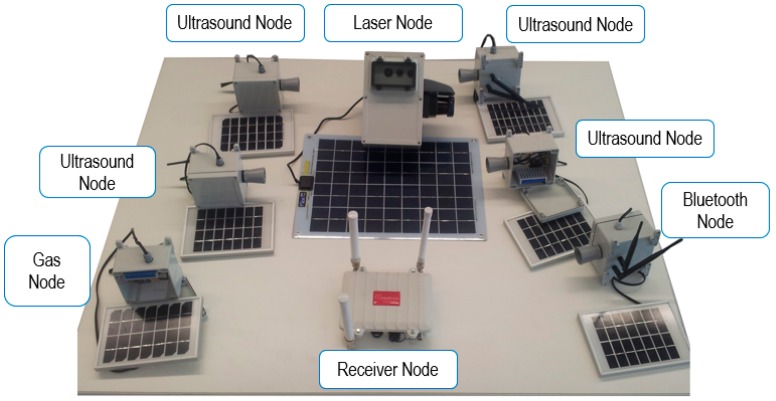
Different types of nodes developed for the Urban Information System.

All transmitter nodes are mounted in IP 67 boxes, so that the system can be deployed outdoors in adverse weather condition. Figure 2 shows different nodes of the UIS platform.

The information gathered from the area of interest is sent to the Receiver node, and stored as tables in its internal database. These tables are synchronized with a relational database implemented with MySQL v.7.0 (Oracle, Redwood City, CA, U.S.A.) in an external server. Synchronization is performed via 3G communication, or Wi-Fi if it is available. This database is the input for an HMI developed for this application using LabVIEW, v. 2015 (National Instruments, Austin, TX, U.S.A.), where the information acquired by the sensors is displayed according to their geographic location. The HMI synchronizes with the external database through a connection string using ODBC v. 13.1 (Microsoft, Redmond, WA, U.S.A.). A more detailed review of the UIS can be found at [7].

## 3. Mobile Node

As seen in Section 1, WSN are a solution for acquiring information close to the area of interest with persistence over time. However, a successful application requires attention to network coverage, connectivity or network overhead. Hybrid wireless sensor networks can provide a solution to some of these challenges. This section presents a mobile node architecture and implementation, and its integration with a static WSN to obtain an H-WSN.

### 3.1. Overview

The UIS is a static WSN. As such, it provides useful information on an area of interest, since it can be deployed with speed and flexibility to adapt to each case requirements. But once the network is deployed, the nodes stay at the same location. Obtaining additional information requires installing supplementary Transmitter nodes, and depending on the distance, also Receiver nodes, due to the limited range of ZigBee communications. Some use-cases require a different approach, such as the study of gas emissions. A significant amount of emissions in urban areas is linked to motor vehicles. Obtaining up-to-date information about the levels of some gases can be very relevant for traffic managers. But deploying a sensor network large enough to cover a whole city might be impractical. A way of obtaining the relevant data that traffic managers require is by means of sensors directly installed on vehicles. But some other applications require also the capability to measure environmental data in an adaptable fast way, such as in disaster robotics, where the collaboration with humans or dogs can be limited by the level of some gases. For those applications requiring additional flexibility in the acquisition of information, a hybrid version of the UIS network has been developed.

These features can be achieved by transforming the static WSN into a H-WSN by adding a Mobile node. The next sections describe the Mobile node architecture and implementation, and its integration with the WSN.

### 3.2. Architecture and Implementation

**Figure 3:**
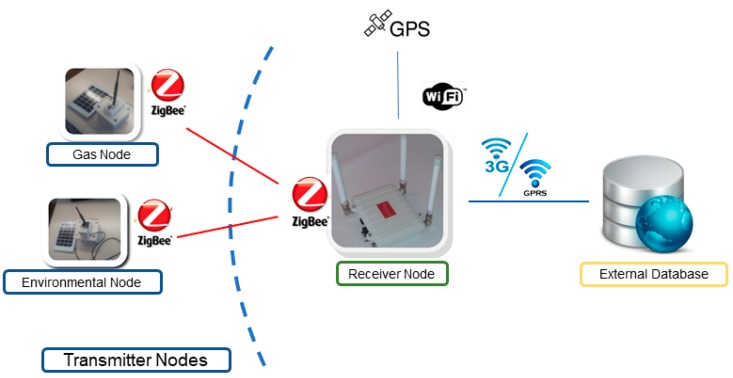
Architecture of the Mobile node.

The Mobile node has been developed to improve the range and flexibility of the UIS. It has been designed to allow installation onboard vehicles, so the area under study can be modified without the need to re-deploy the UIS nodes. A modular architecture was desirable so that the set of sensors could be modified according to mission needs. At the same time, one of the requirements was to use the original nodes with as few modifications as possible. But since the Transmitter nodes send their data using the ZigBee protocol, they have a limited range, which is affected by the obstacles between emitter and receiver. To overcome this limitation, one possibility is to configure ZigBee nodes as routers, allowing multi-hop routing to take the data from the Transmitter nodes to the Receiver node. However, this option limits the flexibility of a mobile node, since it could only move around the position of already deployed nodes. To meet all the requirements a modular architecture was designed including a Receiver node and a configurable number of standard UIS Transmitter nodes, so that no hardware changes are required. The Receiver node gathers the information from the Transmitter nodes, and synchronizes it with the database in the external server via 3G or Wi-Fi (see Figure 3). In this way, the Mobile node can acquire data from areas without the need for previously deployed ZigBee nodes between the Receiver node and the area of interest.

An example set of nodes included in the Mobile node is shown in Figure 3. In this case, a Gas node and an Environmental node are included as the Transmitter nodes, together with the Receiver node. It is worth notice that Transmitter nodes may contain several sensors, which can be changed from one experiment to another depending on the mission requirements. For instance, the Gas node can be equipped with different sets of gas sensors, including CO_2_, CO, O_2_, VOC or NH_3_.

The Mobile node has two possible working modes: local mode and networked mode. In both modes, the Transmitter nodes acquire and process data from the environment, and send them to the embarked Receiver node. The modes differ in the way that a frame is constructed prior to store it in the local table, and to synchronize it with the external database. The implementation had to meet the requirement of using the original nodes with as few modifications as possible. Thus, changes have been limited to the Receiver node software. These modifications have consisted in the creation of two additional software configurations according to the two different working modes. This software configuration has to be selected offline, previously to the start of an experiment. Thus, it is possible to increase the communication range of the existing sensor nodes without the need for integration of a new radio segment with the Transmitter nodes, or the development of new connection strings with the external server according to this new communication link. At the same time, it is possible to use any type of Transmitter node already in use by the static network.

**Figure 4:**
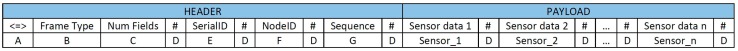
ASCII frame structure employed in the mobile node.

Both working modes use the same frame structure. Within the different frame types for the ZigBee protocol, an ASCII frame structure has been selected. The frame structure is shown in Figure 4, where:

(A) Start delimiter [3 bytes]: It is made by three characters “<=>”.

(B) Frame-type indicator byte [1 byte]: This field defines if the frame is binary (0 × 00) or ASCII form (0 × 80).

(C) Number of sensor fields [1 byte]: It specifies the number of sensor fields included in the frame, in hexadecimal.

(D) Separator [1 byte]: The start of each field is marked by a character “#”, as well as the end of the frame.

(E) SerialID [10 Bytes]: This field is made by a 10-bit-long numeric string which identifies uniquely the device. 

(F) NodeID [0–16 bytes]: String of characters labelling the node. 

(G) Frame sequence [1–3 bytes]: It indicates the sequence number of any frame sent. It is made by 8 bits and numbered from 0 to 255. When the value 255 is reached, the counter resets to 0. Every node has its own frame count.

Sensor_i: Specific field of each sensor including an identifying label and the data that it provides. Its length can be variable. 

#### 3.2.1. Local Mode

In this mode, the Mobile node is seen by the network as a single, multi-sensor node. To do so, an enhanced frame is constructed containing the information from all the sensors present in the Transmitter nodes within the Mobile node. 

**Figure 5:**
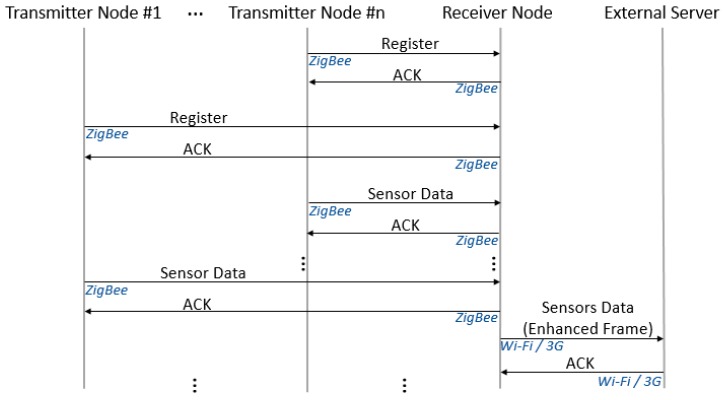
Simplified protocol message flow diagram for the local mode.

Once data from all the sensors have been received, the Receiver node builds an enhanced frame with data from all of them, includes location data and updates the internal database. Synchronization is then performed with the external database (via 3G or Wi-Fi, if available), where the information appears as data from a single node. A simplified protocol message flow diagram for this working mode is shown in Figure 5.

**Figure 6:**
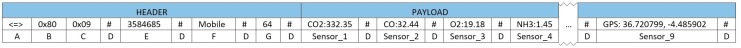
Part of an enhanced frame.

Figure 6 shows an example of part of an enhanced frame built integrating information from different Transmitter nodes. In this case, a Gas node including several gas sensors is installed. Location data are included by the Receiver node in an additional sensor field.

The local mode limits the communications, but only in the 3G/Wi-Fi segment. Thus, the number of data available at the external database is reduced: since a frame is updated in the database only when all Transmitter nodes have sent their data, the dynamics of the data acquisition is as slow as the slowest sensor present. Transmitter nodes keep their own pace sending their data to the Receiver node, but only the most recent data from every sensor are used to construct the enhanced frame. Depending of the types of nodes, real-time presentation of the data might be difficult to attain.

#### 3.2.2. Networked Mode

**Figure 7:**
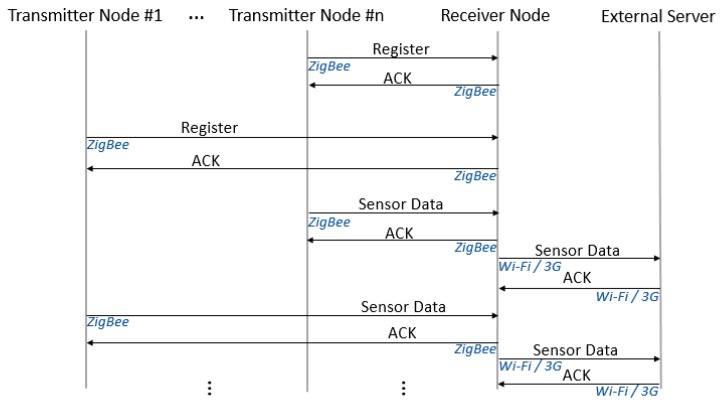
Simplified protocol message flow diagram for the networked mode.

**Figure 8:**
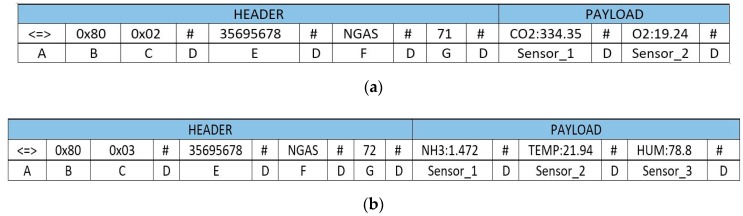
Frame in networked mode with gas sensors data: (**a**) Frame with data from two sensors. (**b**) Frame with data from three sensors.

In the networked mode, when data from any of the Transmitter nodes are available, a frame is constructed and sent to the embarked Receiver node. A frame may contain information from one or several sensors from the same Transmitter node. The Receiver node updates its internal database, and then synchronization with the external database takes place via 3G, or Wi-Fi if it is available. A simplified protocol message flow diagram for this working mode is shown in Figure 7. Figure 8 shows examples of frames with data from a Gas node. In Figure 8a, the frame includes CO_2_ and O_2_ measurements. In Figure 8b, the frame contains measurements from three sensors: NH_3_, temperature and humidity. Location is obtained by means of a GPS node, acting as an additional Transmitter node. This way, more positioning data are available.

This working mode makes available a larger amount of data, through a higher number of updates with the external database. Data are also available to the user faster, since the synchronization of the databases takes place as soon as new data are collected. 

### 3.3. Integration with the H-WSN

**Figure 9:**
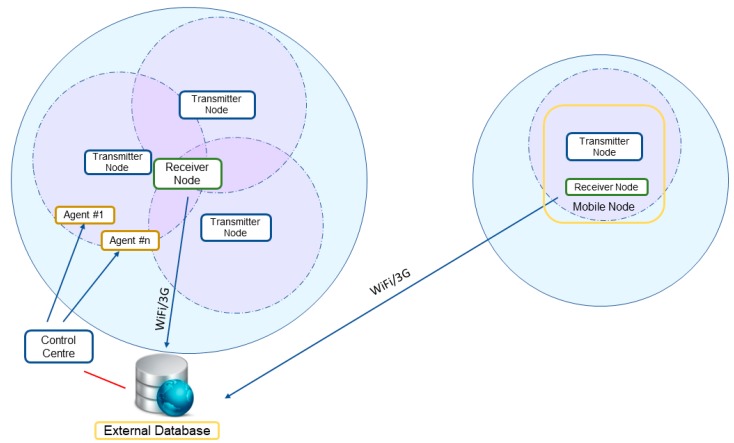
Integration of the Mobile node to create a Hybrid-Wireless Sensor Network.

The data acquired by the sensors on the Mobile node are available to any user through the database, like the data from any other sensor in the static network. The Wireless Sensor Network is then transformed into a Hybrid Wireless Sensor Network (H-WSN), managing data from both static and mobile nodes indifferently. Figure 9 shows the integration of the Mobile node (in the right-hand side) with a static WSN (in the left-hand side), resulting in a H-WSN. The Receiver node acts as a mobile sink, granting coverage for the Transmitter nodes whenever 3G or Wi-Fi coverage is available without the need for multi-hop routing or dynamic relocation methods, which can reduce operating lifetime of the nodes due to increased communication. By transforming the WSN into H-WSN the resulting system gains flexibility, since a Mobile node can be deployed to obtain data from an area not covered by static nodes (for instance, in cases where some pieces of evidence make the area worth studying after the original deployment of the network). Robustness can also be increased with the use of a Mobile node to substitute malfunctioning static nodes. 

**Figure 10:**
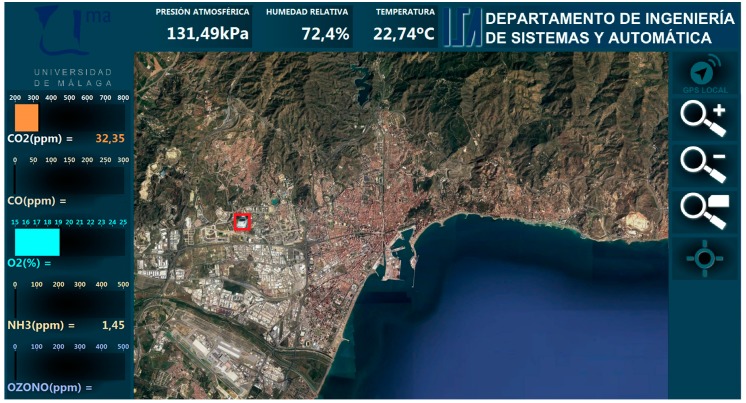
Human–Machine Interface for the Urban Information System.

The external database is the input for an HMI developed for this application using LabVIEW (see Figure 10), making possible to present the information obtained by the different sensors, and related to their locations. The user can configure what sensors’ information is shown, according to the deployed nodes, including static nodes. For instance, Figure 10 shows how the user can see the available measurements for CO_2_, CO, O_2_, NH_3_, ozone (O_3_), atmospheric pressure, relative humidity and temperature, at the beginning of a route. 

The data gathered from the Transmitter nodes are presented to the user associated to their locations. In the case of the Mobile node this location is dynamic, but from the point of view of the WSN it is presented as another node. The HMI has been designed to be compatible with ruggedized tablets running Windows 7. This way, users can also move, either with the Mobile node, or on another vehicle.

The use-case in this article is based on measuring gas levels in an urban scenario, but some other applications are feasible. The Mobile node has been designed to accept as many types of Transmitter nodes as possible, without any modifications in hardware. The Mobile node provides an interface with the network, via an additional Receiver node, and power supply by using a battery bank. Although power supply by photovoltaic panels is still possible, depending on the application it might be unpractical. So far, only the Laser node is to be integrated in the mobile node. This flexibility in the configuration of the sensor suite allows for adaptation to other applications through the modification of the set of sensors and the selection of the platform to carry the Mobile node, allowing for collaborative efforts towards Cyber-Physical System integrating sensors, vehicles or humans. For instance, search and rescue applications present opportunities for this approach [60]. 

## 4. Experiments

**Figure 11:**
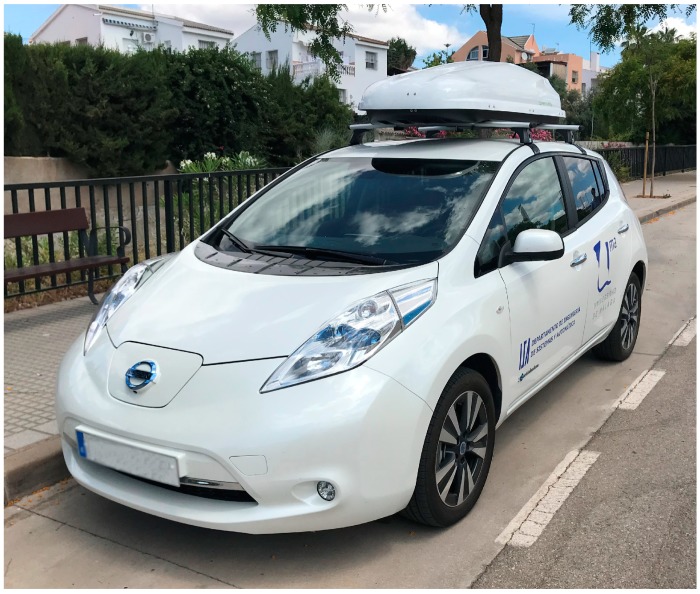
Vehicle used in experiments showing the top case with the UIS Mobile node installed.

A series of experiments has been designed to validate the concept of mobile node. This validation includes using different combinations of nodes and sensors. The experiments have been performed for the two working modes: local and networked. The mobile node was installed in the modified top carrier of a test vehicle. The selected test vehicle was an electric passenger car (Nissan Leaf, Yokohama, Japan) to prevent perturbation of gas and noise measurements (see Figure 11). The experiments consisted in a series of routes along the city of Malaga, in Spain. The Receiver node within the mobile node synchronized its internal database with the database in an external server. A graphical Human–Machine Interface (HMI), running on a laptop, allowed for monitoring in real-time the gathered data related to their geographic position.

### 4.1. Experiments in Local Mode 

In these experiments, the mobile node was configured around the Gas and EP nodes, including sensors for gas concentrations and environmental parameters, providing measurements for concentration of O_2_, CO_2_, VOC as well as temperature, humidity, dust, luminance, and noise. GPS data were obtained from the Receiver node. 

The embedded Gas and EP nodes worked acquiring and processing the respective data and sent them to the embarked Receiver node. Once all sensors had provided data, a full set was completed adding localization via the Receiver node’s GPS. The internal database was updated and synchronized with the external database at the server. 

**Figure 12:**
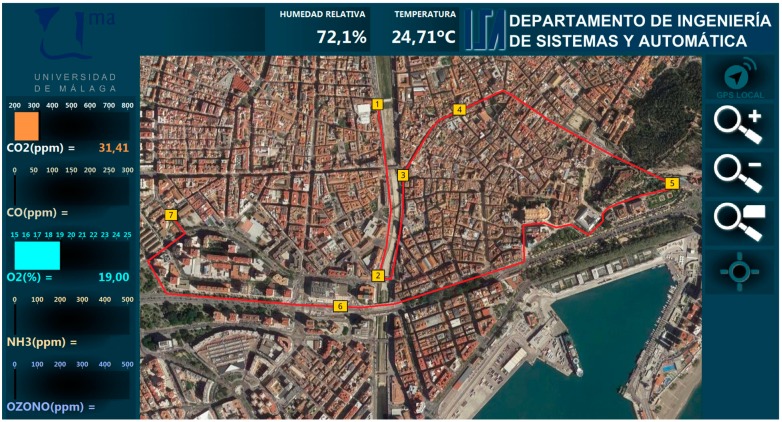
Area of the city center covered by experiments in local mode.

The experiments consisted in deploying the test vehicle along several routes in the city of Malaga. Figure 12 shows a screenshot of the HMI at the end of one of the routes in the city center. The followed path has been signaled with a red line. Numeric labels have been added to mark the locations where an enhanced frame was completed according to the local working mode. This particular experiment had a length of 5 km and took 47 minutes to be completed. 

Table 1 presents the gathered data for the route showed in Figure 12. The item number matches with the labels in the Figure mentioned before. In the local mode, an enhanced frame is constructed once data from all present nodes are acquired, annexing GPS data provided by the Receiver node. Thus, every row in this Table displays the data gathered up to that location. This strategy produces as many frames as the slowest installed sensor. In this particular experiment, the limits were imposed by the dynamics of the CO_2_ sensor. This can be noticed in Figure 12, where locations for frames 1 to 4 are closer than for frames 4 to 7, because the test vehicle could move at a slower velocity in the former, due to traffic jams. Preliminary results from this working mode were described in [58].

### 4.2. Experiments in Networked Mode

In the experiments for the networked mode, the mobile node was configured to include the Gas and GPS nodes. The gas sensors for the former node were NH_3_, O_2_, CO_2_, VOC as well as temperature and humidity. 

The Gas and GPS nodes acquired, processed and sent data to the embarked Receiver node according to their own dynamics. Upon data reception from any sensor, the Receiver node updated its internal database and synchronized it with the external database at the server. GPS data was treated as another sensor, updating the location in the database as soon as new data were received according to a programmed rate.

**Figure 13:**
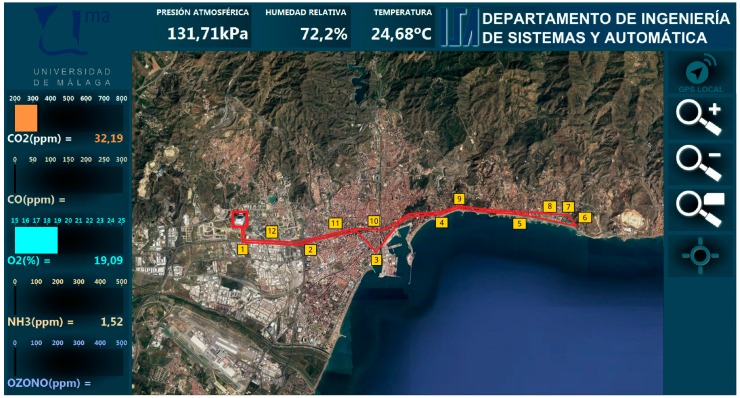
Area of the city of Malaga covered by experiments in networked mode.

**Figure 14:**
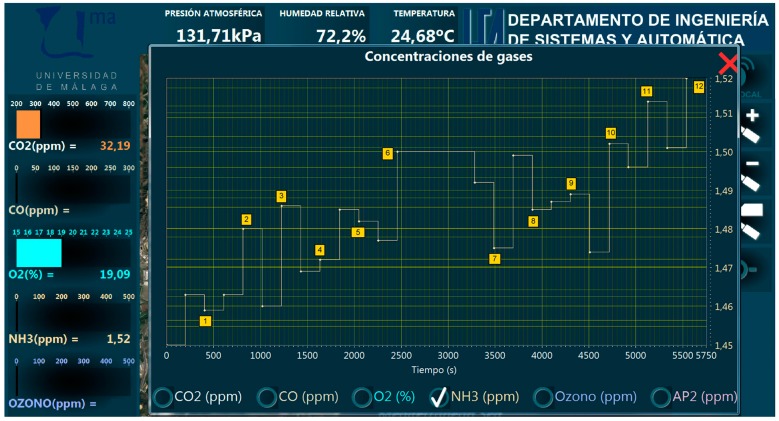
Measurements for the NH3 sensor obtained in a networked mode experiment, as shown by the user interface.

The experiments were designed to consider a greater diversity in the routes, including highways and longer distances. They were performed in the city of Malaga. Figure 13 presents a screenshot of the HMI showing the route of one of these experiments, as well as labels for selected locations. The path started at the campus of the University of Malaga (red square) and reached the eastern limit of the city (label 6) to finally go back to the campus. This route was performed in 1 hour and 24 minutes, for a total of 28.3 kilometers covering highways in the western part of the route (labels 1 and 2) as well as urban areas for the rest. Table 2 presents some of the data obtained by the mobile node together with the geo-located labels in the route marked in Figure 13. The measurements recorded by the different sensors were shown in real-time at the HMI, allowing the user to visualize a plot with the evolution of a certain sensor by selecting it in the interface. Figure 14 shows the evolution of the measurements obtained by the NH_3_ sensor along the route covered by the experiments.

In contrast with the local mode in the networked mode the strategy allows for acquiring a larger amount of data, since the update and synchronization of the databases takes place every time a new data is obtained by any sensor. Thus, the slow dynamics of a sensor does not limit obtaining more data with a faster one. In this way, Table 2 presents a summary of the data, while a more extensive set of measurements is presented in Appendix A. Table 2 presents the last data obtained for every sensor when the test car reached the locations labelled on Figure 13. 

## 5. Conclusions

A modular mobile node has been implemented, capable of using different sensors according to mission needs. The mobile node has been integrated with an existing static network, adding flexibility and range to it and transforming it into a Hybrid Wireless Sensor Network. Integration has avoided the need for multi-hop routing, which can reduce the operating lifetime of the nodes due to increased communication. Two different integration modes have been developed: local mode and networked mode. Data gathered with the mobile node have been presented to the user in real-time, by means of a Human–Machine Interface showing data from a database synchronizing the information gathered by the mobile node, which may include information from other static nodes as well. Finally, the system has been tested in real urban scenarios in a use-case of measurement of gas levels and environmental data.

The tests have comprised both working modes: local and networked. In local mode, the database is updated only after all present Transmitter nodes have sent their data. The dynamics of the Mobile node is then driven by that of the slowest node. Results with the local mode have shown a limited amount of data, as expected. In particular, the slow dynamics of the CO_2_ sensor has produced a low rate of new data arriving to the external database, and then to the user interface. In networked mode, an update of the database takes place every time that a Transmitter node captures data and sends it to the Receiver node. This strategy allows acquisition of a larger amount of data, as evidenced by the experiments carried out. 

The architecture is flexible enough to change easily the number and type of sensors. Experiments have been performed with different sets of sensors, showing no compatibility problems. The modular design of the Mobile node allowed an easy reconfiguration. The flexibility of the system makes it easy to be adapted to other use-cases, like search and rescue missions. The proposed system provides a vehicle with a tailored set of sensors, configured for the needs of the mission. The acquired data are accessible to other vehicles or participants of the mission, allowing collaborative efforts towards a Cyber-Physical System integrating sensors, vehicles or humans. Future lines of work advance in this direction. For instance, an improvement can be planning the motion of the Mobile node according to the acquired data, in order to get an improved coverage or amount of data. This line may be particularly interesting in other use cases, like emergency response. In this case, the set of sensors might be defined according to data gathered by the static part of the WSN, thanks to the modular architecture. Another future line of work includes the study of the robustness of the H-WSN, comparing its performance with a static WSN. Additionally, technologies like LoRaWAN can be an alternative to develop this kind of application. An interesting line is the evaluation of the performance of an alternative implementation based on a different technology, and its comparison with the approach proposed in this paper.

## Figures and Tables

**Table 1:** Data obtained for a city center route in local mode.

Item	EP Node			Gas Node					GPS Coordinates
	Luminance (lux)	Dust (ppm)	Noise (dB)	Temp (°C)	Humidity (%)	O_2_ (%)	CO_2_ (ppm)	VOC (ppm)	
1	25.0	0.074	83	24.00	43.7	19.585	334.300	6.05	36.723861, −4.426139
2	97.0	0.075	93	25.81	47.6	17.794	334.490	5.25	36.717833, −4.426333
3	97.5	0.082	94	23.55	53.2	17.843	334.951	8.69	36.721444, −4.425111
4	98.0	0.074	91	27.10	42.1	18.569	334.615	8.46	36.723722, −4.422750
5	98.5	0.074	91	25.32	51.1	18.133	334.793	7.64	36.721278, −4.413306
6	75.2	0.072	93	27.85	44.3	18.952	334.713	7.94	36.716861, −4.427944
7	98.6	0.074	93	26.29	50.0	18.423	334.694	8.13	36.719722, −4.435972

**Table 2:** Summary of the data obtained by the Mobile node in a route in the city of Málaga, working in networked mode.

Item	NH3 (ppm)	Temp (°C)	Humidity (%)	O_2_ (%)	CO_2_ (ppm)	AP (kPa)	VOC (ppm)	GPS Coordinates
1	1.459	24.19	68.2	18.891	332.310	131.44	2.058	36.713976, −4.482990
2	1.480	22.90	77.4	19.278	332.153	131.83	1.464	36.713168, −4.457434
3	1.486	23.06	77.4	19.182	332.271	132.39	1.598	36.709676, −4.427887
4	1.472	21.94	78.8	19.182	332.193	132.02	1.428	36.720616, −4.403189
5	1.482	22.74	76.1	19.278	331.448	132.07	2.999	36.720149, −4.368288
6	1.500	23.39	74.1	19.182	332.193	132.00	1.428	36.715815, −4.346487
7	1.475	24.03	71.5	18.794	332.114	132.10	1.762	36.720230, −4.349773
8	1.485	26.13	70.7	18.504	332.193	132.24	1.849	36.720692, −4.356785
9	1.489	23.55	71.0	19.182	332.153	132.15	1.273	36.723351, −4.392885
10	1.502	23.71	74.3	19.085	332.114	132.00	1.784	36.717411, −4.423333
11	1.513	25.48	71.5	18.891	331.996	132.41	1.017	36.715752, −4.444772
12	1.519	24.68	72.2	19.085	332.193	131.71	1.103	36.713402, −4.470782

## Appendix A

This appendix presents supplementary data to those discussed in Section 4.2. The table contains all data gathered by the Mobile node between labels 6 and 12 in the experiment shown in 0, showing all the fields in the frames sent to the external server.

**Header**										**Payload**					
**A**	**B**	**C**	**D**	**E**	**D**	**F**	**D**	**G**	**D**	**Sensor_1**	**D**	**Sensor_2**	**D**	**Sensor_3**	**D**
<=>	0x80	0x03	#	387244595	#	NGAS	#	11	#	CO2:331.409	#	NH3:1.492	#	AP:131.83	#
<=>	0x80	0x01	#	387264467	#	NGPS	#	48	#	GPS:36.720272, −4.349771	#				
<=>	0x80	0x01	#	387264467	#	NGPS	#	49	#	GPS:36.720268, −4.349767	#				
<=>	0x80	0x01	#	387264467	#	NGPS	#	50	#	GPS:36.720268, −4.349775	#				
<=>	0x80	0x01	#	387264467	#	NGPS	#	51	#	GPS:36.720245, −4.349782	#				
<=>	0x80	0x01	#	387264467	#	NGPS	#	52	#	GPS:36.720242, −4.349782	#				
<=>	0x80	0x02	#	387244595	#	NGAS	#	12	#	TEMP:24.03	#	HUM:71.5	#		
<=>	0x80	0x02	#	387244595	#	NGAS	#	13	#	O2:18.794	#	VOC:1.76209	#		
<=>	0x80	0x01	#	387264467	#	NGPS	#	53	#	GPS:36.720238, −4.349785	#				
<=>	0x80	0x01	#	387264467	#	NGPS	#	54	#	GPS:36.720238, −4.349807	#				
<=>	0x80	0x01	#	387264467	#	NGPS	#	55	#	GPS:36.720257, −4.349757	#				
<=>	0x80	0x01	#	387264467	#	NGPS	#	56	#	GPS:36.720253, −4.349745	#				
<=>	0x80	0x01	#	387264467	#	NGPS	#	57	#	GPS:36.720230, −4.349773	#				
<=>	0x80	0x03	#	387244595	#	NGAS	#	14	#	CO2:332.114	#	NH3:1.475	#	AP:132.10	#
<=>	0x80	0x01	#	387264467	#	GPS	#	58	#	GPS:36.720230, −4.349777	#				
<=>	0x80	0x01	#	387264467	#	GPS	#	59	#	GPS:36.720249, −4.349757	#				
<=>	0x80	0x01	#	387264467	#	GPS	#	60	#	GPS:36.720257, −4.349753	#				
<=>	0x80	0x01	#	387264467	#	GPS	#	61	#	GPS:36.720257, −4.349753	#				
<=>	0x80	0x01	#	387264467	#	GPS	#	62	#	GPS:36.720257, −4.349760	#				
<=>	0x80	0x01	#	387264467	#	GPS	#	63	#	GPS:36.720257, −4.349745	#				
<=>	0x80	0x01	#	387264467	#	GPS	#	64	#	GPS:36.720257, −4.349743	#				
<=>	0x80	0x02	#	387244595	#	NGAS	#	15	#	TEMP:24.68	#	HUM:71.20	#		
<=>	0x80	0x02	#	387244595	#	NGAS	#	16	#	O2:18.698	#	VOC:1.93993	#		
<=>	0x80	0x01	#	387264467	#	GPS	#	65	#	GPS:36.720257, −4.349741	#				
<=>	0x80	0x01	#	387264467	#	GPS	#	66	#	GPS:36.720242, −4.349741	#				
<=>	0x80	0x01	#	387264467	#	GPS	#	67	#	GPS:36.720242, −4.349797	#				
<=>	0x80	0x01	#	387264467	#	GPS	#	68	#	GPS:36.720249, −4.349783	#				
<=>	0x80	0x01	#	387264467	#	GPS	#	69	#	GPS:36.720383, −4.349657	#				
<=>	0x80	0x03	#	387244595	#	GAS	#	17	#	CO2:332.036	#	NH3:1.499	#	AP:131.95	#
<=>	0x80	0x01	#	387264467	#	GPS	#	70	#	GPS:36.720428, −4.349602	#				
<=>	0x80	0x01	#	387264467	#	GPS	#	71	#	GPS:36.720493, −4.349543	#				
<=>	0x80	0x01	#	387264467	#	GPS	#	72	#	GPS:36.719578, −4.350820	#				
<=>	0x80	0x01	#	387264467	#	GPS	#	73	#	GPS:36.719494, −4.350966	#				
<=>	0x80	0x01	#	387264467	#	GPS	#	74	#	GPS:36.719494, −4.350998	#				
<=>	0x80	0x01	#	387264467	#	GPS	#	75	#	GPS:36.719501, −4.350993	#				
<=>	0x80	0x01	#	387264467	#	GPS	#	76	#	GPS:36.719570, −4.351252	#				
<=>	0x80	0x02	#	387244595	#	NGAS	#	18	#	TEMP:26.13	#	HUM:70.7	#		
<=>	0x80	0x02	#	387244595	#	NGAS	#	19	#	O2:18.504	#	VOC:1.84927	#		
<=>	0x80	0x01	#	387264467	#	GPS	#	77	#	GPS:36.719559, −4.351247	#				
<=>	0x80	0x01	#	387264467	#	GPS	#	78	#	GPS:36.720085, −4.352318	#				
<=>	0x80	0x01	#	387264467	#	GPS	#	79	#	GPS:36.720253, −4.353889	#				
<=>	0x80	0x01	#	387264467	#	GPS	#	80	#	GPS:36.720692, −4.356785	#				
<=>	0x80	0x03	#	387244595	#	NGAS	#	20	#	CO2:332.193	#	NH3:1.485	#	AP:132.24	#
<=>	0x80	0x01	#	387264467	#	NGPS	#	81	#	GPS:36.720848, −4.357680	#				
<=>	0x80	0x01	#	387264467	#	NGPS	#	82	#	GPS:36.721104, −4.360242	#				
<=>	0x80	0x01	#	387264467	#	NGPS	#	83	#	GPS:36.721096, −4.360897	#				
<=>	0x80	0x01	#	387264467	#	NGPS	#	84	#	GPS:36.721336, −4.362478	#				
<=>	0x80	0x02	#	387244595	#	NGAS	#	21	#	TEMP:23.87	#	HUM:70.3	#		
<=>	0x80	0x02	#	387244595	#	NGAS	#	22	#	O2:19.085	#	VOC:1.42802	#		
<=>	0x80	0x01	#	387264467	#	GPS	#	85	#	GPS:36.721630, −4.363220	#				
<=>	0x80	0x01	#	387264467	#	GPS	#	86	#	GPS:36.722065, −4.364815	#				
<=>	0x80	0x01	#	387264467	#	GPS	#	87	#	GPS:36.722317, −4.366760	#				
<=>	0x80	0x01	#	387264467	#	GPS	#	88	#	GPS:36.722363, −4.368630	#				
<=>	0x80	0x01	#	387264467	#	GPS	#	89	#	GPS:36.722301, −4.372442	#				
<=>	0x80	0x03	#	387244595	#	NGAS	#	23	#	CO2:332.114	#	NH3:1.487	#	AP:132.02	#
<=>	0x80	0x01	#	387264467	#	GPS	#	90	#	GPS:36.722355, −4.374267	#				
<=>	0x80	0x01	#	387264467	#	GPS	#	91	#	GPS:36.722115, −4.378480	#				
<=>	0x80	0x01	#	387264467	#	GPS	#	92	#	GPS:36.721836, −4.384040	#				
<=>	0x80	0x01	#	387264467	#	GPS	#	93	#	GPS:36.722389, −4.387465	#				
<=>	0x80	0x02	#	387244595	#	NGAS	#	24	#	TEMP:23.55	#	HUM:71.0	#		
<=>	0x80	0x02	#	387244595	#	NGAS	#	25	#	O2:19.182	#	VOC:1.27315	#		
<=>	0x80	0x01	#	387264467	#	GPS	#	94	#	GPS:36.722607, −4.388041	#				
<=>	0x80	0x01	#	387264467	#	GPS	#	95	#	GPS:36.722618, −4.387952	#				
<=>	0x80	0x01	#	387264467	#	GPS	#	96	#	GPS:36.722633, −4.388866	#				
<=>	0x80	0x01	#	387264467	#	GPS	#	97	#	GPS:36.722767, −4.390242	#				
<=>	0x80	0x01	#	387264467	#	GPS	#	98	#	GPS:36.723351, −4.392885	#				
<=>	0x80	0x03	#	387244595	#	NGAS	#	26	#	CO2:332.153	#	NH3:1.489	#	AP:132.15	#
<=>	0x80	0x01	#	387264467	#	GPS	#	99	#	GPS:36.723610, −4.394613	#				
<=>	0x80	0x01	#	387264467	#	GPS	#	100	#	GPS:36.723675, −4.395510	#				
<=>	0x80	0x01	#	387264467	#	GPS	#	101	#	GPS:36.723385, −4.396703	#				
<=>	0x80	0x01	#	387264467	#	GPS	#	102	#	GPS:36.722881, −4.398278	#				
<=>	0x80	0x02	#	387244595	#	NGAS	#	27	#	TEMP:24.68	#	HUM:72.2	#		
<=>	0x80	0x02	#	387244595	#	NGAS	#	28	#	O2:19.085	#	VOC:1.37480	#		
<=>	0x80	0x01	#	387264467	#	GPS	#	103	#	GPS:36.722881, −4.398252	#				
<=>	0x80	0x01	#	387264467	#	GPS	#	104	#	GPS:36.722195, −4.400375	#				
<=>	0x80	0x01	#	387264467	#	GPS	#	105	#	GPS:36.721539, −4.403050	#				
<=>	0x80	0x01	#	387264467	#	GPS	#	106	#	GPS:36.721138, −4.406835	#				
<=>	0x80	0x03	#	387244595	#	NGAS	#	29	#	CO2:332.114	#	NH3:1.474	#	AP:132.27	#
<=>	0x80	0x01	#	387264467	#	GPS	#	107	#	GPS:36.720963, −4.408802	#				
<=>	0x80	0x01	#	387264467	#	GPS	#	108	#	GPS:36.720982, −4.409337	#				
<=>	0x80	0x01	#	387264467	#	GPS	#	109	#	GPS:36.720963, −4.409273	#				
<=>	0x80	0x01	#	387264467	#	GPS	#	110	#	GPS:36.720985, −4.411543	#				
<=>	0x80	0x01	#	387264467	#	GPS	#	111	#	GPS:36.720058, −4.414030	#				
<=>	0x80	0x02	#	387244595	#	NGAS	#	30	#	TEMP:23.71	#	HUM:74.3	#		
<=>	0x80	0x02	#	387244595	#	NGAS	#	31	#	O2:19.085	#	VOC:1.78357			
<=>	0x80	0x01	#	387264467	#	GPS	#	112	#	GPS:36.719753, −4.415108	#				
<=>	0x80	0x01	#	387264467	#	GPS	#	113	#	GPS:36.718822, −4.418522	#				
<=>	0x80	0x01	#	387264467	#	GPS	#	114	#	GPS:36.717731, −4.422348	#				
<=>	0x80	0x01	#	387264467	#	GPS	#	115	#	GPS:36.717411, −4.423333	#				
<=>	0x80	0x03	#	387244595	#	NGAS	#	32	#	CO2:332.114	#	NH3:1.502	#	AP:132.00	#
<=>	0x80	0x01	#	387264467	#	GPS	#	116	#	GPS:36.717415, −4.423338	#				
<=>	0x80	0x01	#	387264467	#	GPS	#	117	#	GPS:36.717350, −4.423276	#				
<=>	0x80	0x01	#	387264467	#	GPS	#	118	#	GPS:36.717258, −4.423769	#				
<=>	0x80	0x01	#	387264467	#	GPS	#	119	#	GPS:36.717133, −4.424300	#				
<=>	0x80	0x01	#	387264467	#	GPS	#	120	#	GPS:36.716770, −4.425715	#				
<=>	0x80	0x01	#	387264467	#	GPS	#	121	#	GPS:36.716656, −4.427165	#				
<=>	0x80	0x02	#	387244595	#	NGAS	#	33	#	TEMP:25.81	#	HUM:73.8	#		
<=>	0x80	0x02	#	387244595	#	NGAS	#	34	#	O2:19.085	#	VOC:1.24064	#		
<=>	0x80	0x01	#	387264467	#	GPS	#	122	#	GPS:36.716648, −4.427457	#				
<=>	0x80	0x01	#	387264467	#	GPS	#	123	#	GPS:36.716595, −4.428807	#				
<=>	0x80	0x01	#	387264467	#	GPS	#	124	#	GPS:36.716644, −4.428792	#				
<=>	0x80	0x01	#	387264467	#	GPS	#	125	#	GPS:36.716644, −4.428807	#				
<=>	0x80	0X03	#	387244595	#	NGAS	#	35	#	CO2:332.114	#	NH3:1.496		AP:132.54	#
<=>	0x80	0x01	#	387264467	#	GPS	#	126	#	GPS:36.716621, −4.429085	#				
<=>	0x80	0x01	#	387264467	#	GPS	#	127	#	GPS:36.716965, −4.430676	#				
<=>	0x80	0x01	#	387264467	#	GPS	#	128	#	GPS:36.717220, −4.434623	#				
<=>	0x80	0x01	#	387264467	#	GPS	#	129	#	GPS:36.717152, −4.437274	#				
<=>	0x80	0x02	#	387244595	#	NGAS	#	36	#	TEMP:25.48	#	HUM:71.5	#		
<=>	0x80	0x02	#	387244595	#	NGAS	#	37	#	O2:18.891	#	VOC:1.01747	#		
<=>	0x80	0x01	#	387264467	#	GPS	#	130	#	GPS:36.716610, −4.440198	#				
<=>	0x80	0x01	#	387264467	#	GPS	#	131	#	GPS:36.716297, −4.441992	#				
<=>	0x80	0x01	#	387264467	#	GPS	#	132	#	GPS:36.716305, −4.442037	#				
<=>	0x80	0x01	#	387264467	#	GPS	#	133	#	GPS:36.715752, −4.444772	#				
<=>	0x80	0x03	#	387244595	#	NGAS	#	38	#	CO2:331.996	#	NH3:1.513	#	AP:132.41	#
<=>	0x80	0x01	#	387264467	#	GPS	#	134	#	GPS:36.715527, −4.446649	#				
<=>	0x80	0x01	#	387264467	#	GPS	#	135	#	GPS:36.715346, −4.447702	#				
<=>	0x80	0x01	#	387264467	#	GPS	#	136	#	GPS: 36.714713, −4.449996	#				
<=>	0x80	0x01	#	387264467	#	GPS	#	137	#	GPS: 36.714381, −4.451124	#				
<=>	0x80	0x02	#	387244595	#	NGAS	#	39	#	TEMP:25.21	#	HUM:71.7	#		
<=>	0x80	0x02	#	387244595	#	NGAS	#	40	#	O2:18.982	#	VOC:1.31624	#		
<=>	0x80	0x01	#	387264467	#	GPS	#	138	#	GPS: 36.714170, −4.452328	#				
<=>	0x80	0x01	#	387264467	#	GPS	#	139	#	GPS: 36.713809, −4.453980	#				
<=>	0x80	0x01	#	387264467	#	GPS	#	140	#	GPS: 36.713417, −4.455783	#				
<=>	0x80	0x01	#	387264467	#	GPS	#	141	#	GPS: 36.712994, −4.457854	#				
<=>	0x80	0X03	#	387244595	#	NGAS	#	41	#	CO2:332.057	#	NH3:1.511		AP:132.12	#
<=>	0x80	0x01	#	387264467	#	GPS	#	142	#	GPS: 36.712782, −4.459396	#				
<=>	0x80	0x01	#	387264467	#	GPS	#	143	#	GPS: 36.712571, −4.460751	#				
<=>	0x80	0x01	#	387264467	#	GPS	#	144	#	GPS: 36.712601, −4.462669	#				
<=>	0x80	0x01	#	387264467	#	GPS	#	145	#	GPS: 36.712782, −4.465112	#				
<=>	0x80	0x02	#	387244595	#	NGAS	#	42	#	TEMP:24.68	#	HUM:72.2	#		
<=>	0x80	0x02	#	387244595	#	NGAS	#	43	#	O2:19.085	#	VOC:1.10321	#		
<=>	0x80	0x01	#	387264467	#	GPS	#	146	#	GPS: 36.712992, −4.466951	#				
<=>	0x80	0x01	#	387264467	#	GPS	#	147	#	GPS: 36.713233, −4.469393	#				
<=>	0x80	0x01	#	387264467	#	GPS	#	148	#	GPS: 36.713413, −4.471533	#				
<=>	0x80	0x01	#	387264467	#	GPS	#	149	#	GPS: 36.713402, −4.470782	#				
<=>	0x80	0x03	#	387244595	#	NGAS	#	44	#	CO2:332.193	#	NH3:1.519	#	AP:131.71	#
